# Association of Periodontal Disease and Tooth Loss with Cancer in Korean Adults: A Nationwide Cross-Sectional Study

**DOI:** 10.3290/j.ohpd.c_2151

**Published:** 2025-08-04

**Authors:** Eun-Seo Jung, Hae-Young Kim

**Affiliations:** a Eun-Seo Jung Transdisciplinary Major in Learning Health Systems, Department of Public Health Science, Graduate School, Korea University, Seoul, Korea. Conceptualisation, methodology, formal analysis, writing – original draft, writing – review and editing.; b Hae-Young Kim Transdisciplinary Major in Learning Health Systems, Department of Public Health Science, Graduate School, Korea University, Seoul, Korea; Department of Health Care Sciences, Graduate School, Korea University, Seoul, Korea; Department of Health Policy and Management, College of Health Science, Korea University, Seoul, Korea. Conceptualisation, methodology, supervision, writing – review and editing.

**Keywords:** cancer, Korea National Health and Nutrition Examination Survey, oral health, periodontal disease, tooth loss

## Abstract

**Purpose:**

The precise mechanisms underlying cancer development remain unclear, and limited research has been conducted on the association between periodontal health and cancer in the Korean population. The aim of this study was to evaluate the relationship between periodontal status and tooth loss with the cancer risk among Korean adults by using data from the Korea National Health and Nutrition Examination Survey (2010–2018).

Materials and Methods: This cross-sectional study included 13,616 adults aged ≥ 19 years who participated in health surveys and oral examinations. Logistic regression analysis was conducted to evaluate the associations of periodontal status (community periodontal index [CPI]) and tooth loss with cancer. We adjusted the model for demographic, health-related, and oral health-related factors in a stepwise fashion to minimise confounding variables.

**Results:**

Participants with a CPI score of 3–4 (indicating periodontitis) had slightly higher odds of having cancer in minimally adjusted models than those with a CPI score of 0, but this association was not significant after full adjustment. Conversely, individuals with eight or more missing teeth showed consistently higher odds of having cancer than those with no missing teeth across all models, even after adjusting for confounders (adjusted odds ratio: 1.23; 95% CI: 1.11–1.73).

**Conclusion:**

The findings suggest that tooth loss may be a stronger indicator of cancer risk than periodontal disease. Further studies with longitudinal designs are needed to clarify the causal relationships between oral health and cancer development.

In Korea, cancer remains a leading cause of mortality, with 254,718 cases reported in 2019 and a mortality rate of 161.1 per 100,000 people in 2021.^22,23^ Chronic inflammation has been identified as a critical factor in cancer development, as it weakens the immune system and contributes to malignancy.^17,19^ Periodontal disease, characterised by chronic inflammation caused by bacterial plaque, has been proposed as a potential risk factor for cancer owing to its systemic effects beyond the oral cavity, possibly influencing tissues and organs through inflammatory mediators.^4,7,15^ Recent reviews have further reinforced the biological plausibility of the link between periodontal inflammation and cancer, particularly through mechanisms involving systemic inflammatory mediators and host immune responses.^27,30^

While the relationship between periodontal disease and cancer has been discussed in previous studies, evidence supporting causality remains insufficient.^9,18,24^ Most existing studies were conducted on populations with different genetic and cultural backgrounds, limiting their applicability to Korean adults.^3,10,20^ Moreover, researchers have often failed to comprehensively consider confounding factors or to analyse periodontal status and tooth loss as separate but potentially related risk factors.^19,24^ Given that tooth loss may reflect cumulative exposure to poor oral health or systemic conditions over time, it warrants further investigation alongside periodontal status.^10,20^ Therefore, using data from the nationally representative Korea National Health and Nutrition Examination Survey (KNHANES; 2010–2018), we conducted this study with the aim to evaluate the direct association of periodontal status and tooth loss with cancer among Korean adults while controlling for key confounding variables. We hypothesised that periodontal disease and tooth loss are significantly associated with an increased risk of cancer in Korean adults, even after controlling for major confounding factors.

## MATERIALS AND METHODS

This study adhered to the STROBE guidelines (www.strobe-statement.org) for the reporting of observational studies. Data were obtained from the KNHANES, a publicly available, nationally representative cross-sectional survey designed to evaluate the health and nutritional status of the Korean population. As we used publicly accessible data for this study, the requirement for written informed consent was waived. Ethical approval was obtained from the Institutional Review Board of the Korea Centers for Disease Control and Prevention (approval number: XXX), and the study was conducted in accordance with the 2002 version of the World Medical Association Declaration of Helsinki.

**Fig 1:**
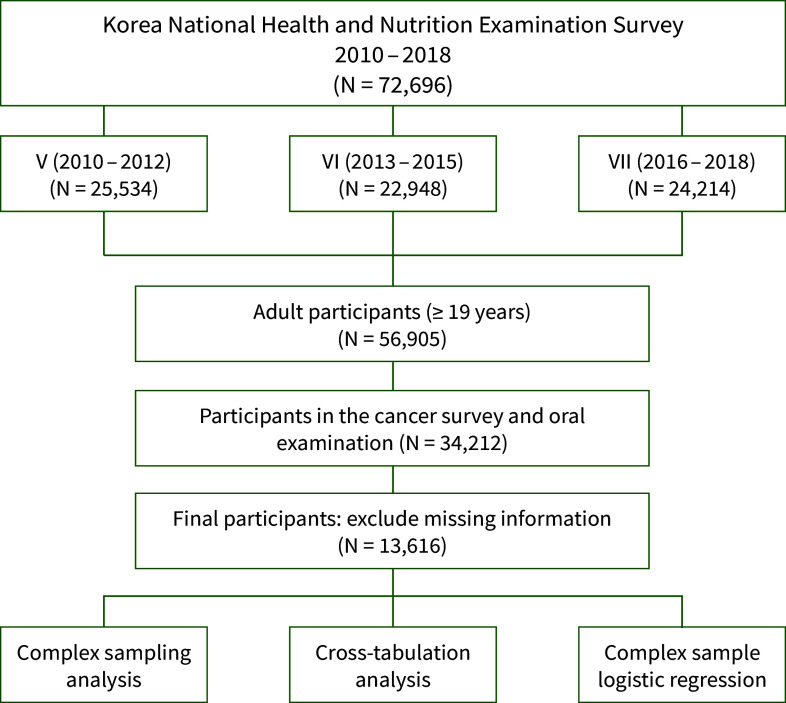
Flow diagram showing selection of the study sample.

The analysis utilised data from the fifth (2010–2012), sixth (2013–2015), and seventh (2016–2018) KNHANES cycles. Of the 72,696 participants during this period, individuals aged ≥19 years were selected. After excluding participants with missing data for key variables, the final sample comprised 13,616 participants (Fig 1).

### Variables and Measurements

Demographic variables included sex and age, whereas socioeconomic variables comprised educational attainment (elementary school graduation or below, middle school graduation, high school graduation, and university graduation or above) and household income (low, lower-middle, upper-middle, and high quartiles). Health behavioural factors included smoking status (current smokers, former smokers, or never-smokers) and alcohol consumption (less than once a month or at least once a month). Oral health behavioural factors included tooth brushing frequency (<3 times/day or ≥3 times/day) and dental check-up history in the past year (yes or no).

Periodontal status was assessed by trained dentists using the community periodontal index (CPI). The examination focused on six sites in the oral cavity: the maxillary and mandibular first and second molars, and the maxillary right and mandibular left central incisors. Periodontal disease was defined as a probing depth of the periodontal pocket ≥4 mm. CPI scores ranged from 0 (healthy) to 4 (deep periodontal pocket), and the highest score among the six sites was used as the representative value. Participants were categorised into three groups for analysis: normal (CPI = 0), gingivitis (CPI = 1–2), and periodontitis (CPI = 3–4). The number of missing teeth was calculated based on 28 teeth, excluding the third molars.

Cancer diagnosis was determined based on self-reported responses to the question, ‘Have you ever been diagnosed with cancer by a physician?’ Participants were categorised as ‘cancer’ or ‘non-cancer’ based on their responses. Details regarding cancer stage, treatment, and recurrence were not available. Cancers included gastric, liver, colorectal, lung, thyroid, and other cancers. Sex-specific cancers were excluded due to their distinct hormonal and biological characteristics, which may involve different pathophysiological mechanisms compared to cancers commonly associated with chronic inflammation and oral health status.

### Statistical Analysis

To account for the complex sampling design of the KNHANES, we applied sampling weights, cluster extraction variables, and variance estimation strata for all analyses. Chi-square tests were used to assess differences in cancer prevalence across participant characteristics. Logistic regression models were employed to evaluate the association of periodontal status and tooth loss with cancer. Multivariable logistic regression models were adjusted for confounding variables in a stepwise manner: (a) sex, age, education level, and household income; (b) additional adjustment for smoking status and alcohol consumption; and (c) further adjustment for tooth brushing frequency and dental check-up history in the past year. Odds ratios (ORs) and 95% confidence intervals (CIs) were calculated, and statistical significance was set at P <0.05. All analyses were conducted using SAS version 9.4 (SAS Institute, Cary, NC, USA).

## RESULTS

The weighted prevalence of cancer and periodontitis in the overall sample was 2.7% (95% CI: 2.4–2.9%) and 28.5% (95% CI: 27.1–29.9%), respectively. Participants diagnosed with cancer were older, had lower educational and income levels, and were more likely to have been past smokers or to consume alcohol less frequently than those without cancer. Additionally, individuals who brushed their teeth fewer than three times per day or had periodontitis exhibited a higher prevalence of cancer than their counterparts. The average number of missing teeth was significantly greater among participants diagnosed with cancer than among those without cancer (Table 1).

**Table 1:** Demographic distribution according to cancer diagnosis

Characteristic	Division	Total	Cancer	Non-cancer	P^1^
(n = 13,616)	(n = 460)	(n = 13,156)
Sex	Male	5,737 (49.1)	228 (2.8)	5,509 (97.2)	0.306
Female	7,879 (50.9)	232 (2.5)	7,647 (97.5)
Age, years	Mean ± SD	45.32 ± 0.23	60.01 ± 0.88	44.91 ± 0.23	**<0.001**
19–39	4.060 (39.4)	17 (0.4)	4,043 (99.6)
40–64	6,481 (46.9)	204 (3.0)	6,277 (97.0)
≥65	3,075 (13.7)	239 (7.9)	2,836 (92.1)
Education level	≤Elementary school	2,983 (15.4)	165 (5.4)	2,818 (94.6)	**<0.001**
Middle school	1,451 (9.0)	83 (5.3)	1,368 (94.7)
High school	4,703 (38.7)	127 (2.1)	4,576 (97.9)
≥University or college	4,479 (36.9)	85 (1.5)	4,394 (98.5)
Household income	Low	2,436 (14.4)	132 (4.8)	2,304 (95.2)	**<0.001**
Middle–low	3,464 (24.7)	123 (2.6)	3,341 (97.4)
Middle–high	3,768 (29.8)	101 (2.1)	3,667 (97.9)
High	3,948 (31.1)	104 (2.2)	3,844 (97.8)
Smoking	Never smoker	8,392 (57.3)	254 (2.5)	8,138 (97.5)	**<0.001**
Former smoker	2,708 (20.1)	160 (4.6)	2,548 (95.4)
Current smoker	2,516 (22.6)	46 (1.3)	2,470 (98.7)
Alcohol consumption	<1 per month	6,314 (41.0)	267 (3.4)	6,047 (96.6)	**<0.001**
≥1 per month	7,302 (59.0)	193 (2.1)	7,109 (97.9)
Toothbrushing	<3 per day	6,670 (47.4)	268 (3.2)	6,402 (96.8)	**<0.001**
≥3 per day	6,946 (52.6)	192 (2.2)	6,754 (97.8)
Dental check-up in the past year	Yes	4,077 (30.0)	153 (3.0)	3,924 (97.0)	0.096
No	9,539 (67.0)	307 (2.5)	9,232 (97.5)
Periodontal status	CPI = 0	3,879 (29.8)	121 (2.3)	3,758 (97.7)	**0.002**
CPI = 1–2	5,458 (41.7)	164 (2.3)	5,294 (97.7)
CPI = 3–4	4,279 (28.5)	175 (3.5)	4,104 (96.5)
Number of missing teeth	Mean ± SD	2.59 ± 0.06	5.30 ± 0.37	2.51 ± 0.06	**<0.001**
0	5,955 (50.8)	115 (1.5)	5,840 (98.5)
1–7	5,676 (38.9)	211 (3.1)	5,465 (96.9)
≥8	1,985 (10.3)	134 (6.6)	1,851 (93.4)
Data are presented as the mean ± standard error for continuous variables or as n (%) for categorical variables. Unweighted (n), weighted (%) CPI, community periodontal index. ^1^Chi-square test or t-test.

Table 2 presents the results of the univariate logistic regression analysis. Significant associations with cancer were observed for age, education level, household income, smoking, alcohol consumption, tooth brushing frequency, periodontal status, and the number of missing teeth. Participants with periodontitis had 1.5-times higher odds of having cancer than those with a healthy periodontal status. Additionally, individuals with eight or more missing teeth had 4.5-times higher odds of having cancer than those with no missing teeth.

**Table 2:** Univariate logistic regression analysis for periodontal status and cancer

Characteristic	Division	Cancer	P^1^
Crude OR (95% CI)
Sex	Male	*Ref*	
Female	0.890 (0.712–1.112)	0.306
Age, years	19–39	*Ref*	
40–64	6.681 (3.821–11.683)	**<0.001**
≥65	19.433 (11.012–24.295)	**<0.001**
Education level	≤Elementary school	*Ref*	
Middle school	0.972 (0.701–1.347)	0.862
High school	0.370 (0.281–0.487)	**<0.001**
≥University or college	0.261 (0.188–0.362)	**<0.001**
Household income	Low	*Ref*	
Middle–low	0.539 (0.400–0.725)	**<0.001**
Middle–high	0.432 (0.318–0.587)	**<0.001**
High	0.442 (0.327–0.598)	**<0.001**
Smoking	Never smoker	*Ref*	
Former smoker	1.878 (1.484–2.377)	**<0.001**
Current smoker	1.497 (1.337–1.735)	**<0.001**
Alcohol consumption	<1 per month	*Ref*	
≥1 per month	0.601 (0.481–0.752)	**<0.001**
Toothbrushing	<3 per day	*Ref*	
≥3 per day	0.673 (0.543–0.834)	**<0.001**
Dental check-up in the past year	Yes	*Ref*	
No	0.814 (0.638–1.038)	**0.096**
Periodontal status	CPI = 0	*Ref*	
CPI = 1–2	1.017 (0.755–1.371)	**<0.001**
CPI = 3–4	1.539 (1.159–2.042)	**<0.001**
Number of missing teeth	0	*Ref*	
1–7	2.024 (1.540–2.660)	**<0.001**
≥8	4.490 (3.354–6.010)	**<0.001**
ORs and CIs were calculated using logistic regression analysis.CI, confidence interval; CPI, community periodontal index; OR, odds ratio, Ref, reference. ^1^ Logistic regression.

In the multivariable logistic regression analysis (Table 3), participants with eight or more missing teeth had consistently higher odds of having cancer than those with no missing teeth, with the association remaining significant in the fully adjusted model (model 3; OR = 1.23; 95% CI: 1.11–1.73). However, the association between periodontitis and cancer observed in the minimally adjusted models (models 1 and 2) was not significant in the fully adjusted model (model 3). These findings suggest that tooth loss is a stronger indicator of cancer than periodontal disease when adjusting for key confounding variables.

**Table 3:** Multivariable logistic regression analysis for periodontal status and cancer

**Variables**	**Model 1** ^†^	**P value**	**Model 2** ^‡^	**P value**	**Model 3** ^§^	**P value**
Adjusted OR (95%CI)	Adjusted OR (95%CI)	Adjusted OR (95%CI)
Periodontal status CPI = 0 CPI = 1–2 CPI = 3–4	*Ref* 1.016 (1.010–1.365) 1.225 (1.114–1.474)	**0.032** **0.021**	*Ref* 1.052 (1.023–1.312) 1.101 (1.086–1.436)	**0.026** **0.005**	*Ref* 1.074 (0.890–1.446) 1.012 (0.942–1.198)	**0.054** **0.442**
Number of missing teeth 0 1–7 ≥8	*Ref* 1.020 (1.011–1.341) 1.188 (1.010–1.365)	**0.047** **0.033**	*Ref* 1.035 (1.022–1.374) 1.240 (1.104–1.741)	**0.012** **0.023**	*Ref* 1.017 (0.089–1.354) 1.230 (1.110–1.725)	0.121 **0.035**
ORs and CIs were calculated using logistic regression analysis. CI, confidence interval; CPI, Community Periodontal Index; OR, odds ratio, Ref, reference. ^†^ Adjusted for sex, age, education level, and household income. ^‡^ Further adjusted for smoking and alcohol consumption. ^§^ Further adjustment for tooth brushing and dental check-ups in the past year.

## DISCUSSION

For this study, we utilised nationally representative data from the KNHANES to investigate the association of periodontal status and tooth loss with cancer prevalence. The weighted prevalence of periodontitis and cancer was 28.5% and 2.7%, respectively, consistent with the 2020 KNHANES database for periodontitis prevalence (30.2%) and the National Cancer Information Center report for cancer prevalence (2.7%).^16,23^ In comparison, higher prevalence rates were observed in the US, where the 2020 National Health and Nutrition Examination Survey revealed a periodontitis prevalence of 42%, and the American Cancer Society noted a cancer prevalence of 5% in 2021.^2,5^ Despite these prevalence rates, multivariable logistic regression analysis revealed that tooth loss showed a more consistent association with cancer risk than periodontal disease. This finding underscores the importance of tooth loss as a key indicator of systemic health and its potential role in cancer risk assessment.

The lack of a significant association between periodontal disease and cancer in the fully adjusted models may be explained by confounding variables, such as socioeconomic status, smoking status, and alcohol consumption. For instance, socioeconomic status is known to influence both periodontal health and cancer risk.^17,24^ Although we attempted to minimise confounding effects by adjusting for these variables, residual confounding likely persisted owing to the broad categorisation of variables, such as income. This highlights the need for robust analytical approaches to effectively control for confounding variables in studies of multifactorial diseases such as cancer.^19,24^

In contrast, tooth loss showed a consistently significant association with cancer across all adjusted models. Tooth loss reflects the cumulative effects of chronic inflammation, particularly caused by advanced periodontitis, which may increase the cancer risk through systemic impacts.^4,6^ The significantly greater number of missing teeth among participants with cancer may be explained by several factors. First, tooth loss may result from long-term periodontal inflammation, which contributes to systemic inflammatory responses and potentially cancer development.^19,21.26^ Second, tooth loss may reflect cumulative exposure to unhealthy behaviours or unfavourable socioeconomic conditions – such as smoking, alcohol consumption, or limited access to oral healthcare – that are also known risk factors for cancer.^14,19,24^ Finally, cancer patients may experience oral complications due to immunosuppression or cancer treatments, which can further exacerbate tooth loss.^24^ In light of recent findings, including those highlighting the role of periodontal pathogens, immune dysregulation, and viral infections in cancer development, the association between oral health and systemic disease appears increasingly complex and multifactorial.^26,29^ Previous studies also revealed associations between tooth loss and various cancers, including gastric, colorectal, and lung cancers.^10,13,14^ The findings of this study support these earlier observations, suggesting that tooth loss may serve as a potential indicator of the pathway linking chronic inflammation to cancer development. However, further research is needed to clarify the underlying mechanisms of this association.^4,21^

This study also provides important public health insights. The observed association between oral health and cancer risk highlights the need to address shared risk factors such as unhealthy diet, smoking, and lack of access to preventive care that may contribute to both oral and systemic diseases.^25^ Some countries, such as the United States and Canada, have established systematic management of oral health through early screening, preventive measures, and treatment services.^2,5^ However, in Korea, limited health insurance coverage for dental treatments creates barriers to adequate oral healthcare.^16^ Expanding access to dental treatments and integrating oral health management into overall healthcare policies may reduce the long-term impact of oral inflammation, alleviate the burden of chronic diseases, and improve public health outcomes.^11,12^

This study has several limitations. First, owing to the cross-sectional design of the KNHANES, the causality of the relationships between periodontal status and tooth loss with cancer could not be established.^16^ Second, the cancer diagnoses relied on self-reported data, which might have introduced recall bias. Additionally, the relatively small number of participants diagnosed with cancer in this study limits the generalizability of the findings. Third, the study did not include key risk factors specific to certain types of cancer, such as dietary habits for gastric cancer, occupational exposure for lung cancer, or genetic predispositions for colorectal cancer, which restricts the interpretation of the results.^1,13^ Fourth, although we adjusted for confounding variables, the potential impact of unmeasured confounders cannot be ruled out.^19,24^

Nonetheless, this study makes significant contributions in its analysis of the relationship between oral and systemic health by using 9 years of KNHANES data. KNHANES provides nationally representative and rigorously collected data, making it a robust foundation for evaluation of the relationship between oral health and systemic diseases among Korean adults.^12,16^ Importantly, this study highlights the association between tooth loss and cancer, emphasising the critical role of oral health in systemic health.^8^ The findings suggest that oral health may serve as an indicator of systemic health, offering new perspectives on the broader implications of oral health management.^4,21^

Furthermore, this study provides a unique perspective in its analysis of data from an Asian population, distinguishing it from the predominantly Western-centric research on this topic. By exploring the association between oral health and cancer in a Korean population, it offers valuable insights that can inform national policies on oral healthcare and contribute to global discussions on the importance of oral health.^10,25^ Such region-specific analyses enhance the global understanding of the role of oral health in systemic health and provide practical insights for the development of tailored health policies.^9,14^

Additionally, this study demonstrates that tooth loss, a major consequence of advanced oral diseases, is associated not only with systemic inflammation but also with important systemic diseases, such as cancer. This underscores the need for an integrated public health approach that prioritises the prevention and management of chronic diseases. These findings go beyond the traditional perception of oral diseases as localised issues, linking them to chronic inflammation and systemic health outcomes.^24,28^ These findings suggest that clinicians should pay closer attention to oral health status, particularly tooth loss, as a potential indicator of systemic disease risk. Integrating oral health assessments into general health evaluations may help identify high-risk individuals early and support the development of timely intervention strategies.

Future studies should adopt longitudinal designs and include clinical data to clarify the mechanisms underlying the association between oral health and cancer, as well as to establish the causality of relationships. Given the public health significance of oral health management highlighted by this study, systematic and rigorous epidemiological research is needed to elucidate the bidirectional relationship between oral health and cancer. Such research can inform the development of early intervention and preventive strategies to improve oral health and reduce the burden of systemic diseases. By addressing these challenges, future research can provide actionable insights for the reduction of cancer risk and improvement of overall health outcomes.

In conclusion, this study demonstrates that both periodontal disease and tooth loss are associated with cancer prevalence among Korean adults, with tooth loss showing a more consistent association, suggesting the potential of oral health status as an indicator of systemic disease burden.
